# Thermal stability, ligand binding and allergenicity data of Mus m 1.0102 allergen and its cysteine mutants

**DOI:** 10.1016/j.dib.2020.105355

**Published:** 2020-02-29

**Authors:** Elena Ferrari, Romina Corsini, Samuele E. Burastero, Fabio Tanfani, Alberto Spisni

**Affiliations:** a Dept. Medicine and Surgery, University of Parma, via Gramsci 14, 43126 Parma, Italy; b Div. Immunology, IRCCS San Raffaele, Via Olgettina 60, 20132 Milano, Italy; c Dept. Life and Environmental Sciences, Marche Polytechnic University, via Brecce Bianche, 60131 Ancona, Italy

**Keywords:** Lipocalin allergen, Mus m 1 allergen, Thermal stability, Aggregation

## Abstract

The presented data were obtained with the lipocalin allergen Mus m 1.0102 and its cysteine mutants MM-C138A, MM-C157A and MM-C138,157A, whose structural features and unfold reversibility investigations are presented in the research article entitled “The allergen Mus m 1.0102: cysteine residues and molecular allergology” [1].

The data were obtained by means of a Dynamic Light Scattering-based thermal stability assay, a Fluorescence-based ligand-binding assay and a basophil degranulation test, and describe proteins’ fold stability, ligand binding ability and allergenic potential, respectively. Analysis of the collected data produced the temperatures corresponding to the onset of the protein unfolding, the dissociation constants for N-Phenyl-1-naphthylamine ligand and the profiles of β-hexosaminidase release from RBL SX-38 cells, sensitized with the serum of selected allergic patients and incubated with increasing antigens concentrations.

These data allow for comparison of the lipocalin allergen Mus m 1.0102 with its conserved cysteines mutants and, with regard to their potential application in allergy diagnostics and immunotherapy, they contribute to the process of recombinant allergen characterization and standardization.

**Table:** Specifications Table

Subject	Biochemistry, Immunology
Specific subject area	Recombinant allergen characterization
Type of data	Figures, Table
How data were acquired	Dynamic Light Scattering: Malvern Zetasizer μV instrument, controlled by Malvern Zetasizer software Fluorescence: JASCO FP-6200 spectrofluorometer, equipped with a Peltier-controlled cuvette holder and controlled by Spectra Manager™ software package Spectrophotometry: Colibri Microvolume Spectrometer, Titertek-Berthold
Data format	Raw Data
Parameters for data collection	Protein thermal unfolding was carried out using an automated 2.5 °C incremental temperature ramp in the interval 25–80 °C. Binding of the fluorescent probe N-Phenyl-1-naphthylamine to protein samples was monitored by exciting at 337 nm and recording the signal at 401 nm. β-hexosaminidase assay was performed with 4-Nitrophenyl N-acetyl-β-d-galactosaminide substrate, added to sensitized RBL cells supernatants; absorbance was measured at 405 nm.
Description of data collection	Protein light scattering intensities were acquired and used to calculate the temperature of the onset of protein unfolding. Ligand binding data were obtained by direct titration experiments. RBL cells were sensitized with IgE from ten allergic patients and stimulated with allergen increasing concentrations; a dose-response measurement of β-hexosaminidase release was performed.
Data source location	Conformational Data were acquired at Department of Medicine and Surgery, University of Parma, Parma (Italy) Allergenicity Data were acquired at IRCCS San Raffaele Hospital, Immunology Division, Milano (Italy) Allergic Patients origin: Milano (Italy) Longitude of Parma: 10.328000, Latitude of Parma: 44.801472 (decimal degrees) GPS-coordinates of Parma: 44° 48′ 5.2992″ N - 10° 19′ 40.8000″ E Longitude of Milano: 9.1859243, Latitude of Milano: 45.4654219 (decimal degrees) GPS-coordinates of Milano: 45° 27′ 55.519″ N - 9° 11′ 9.327″ E
Data accessibility	The raw data files are provided in Mendeley data repository Data identification number: https://doi.org/10.17632/yscyvryz8t.1 Direct URL to data: https://doi.org/10.17632/yscyvryz8t.1
Related research article	Ferrari E. et al. *The allergen Mus m 1.0102: cysteine residues and molecular allergology* Molecular Immunology, 120 (2020), 1–12 https://doi.org/10.1016/j.molimm.2020.01.022

**Table:** 

**Value of the Data** • The data are useful to describe fold stability, ligand binding ability and allergenic potential of the recombinant lipocalin allergen Mus m 1.0102 and they allow for comparison with its conserved cysteines mutants. • The data highlight the enhanced thermal stability of MM-C138A mutant, without a relevant modification of its binding function and *in vitro* allergenicity. • The data contribute to the process of the recombinant allergen standardization, focused to its potential use in immunotherapy and diagnostics applications. • Because of the high grade of conservation of lipocalin cysteines, these data suggest that a similar analytical approach might be applied for the preliminary characterization of any recombinant lipocalin allergen and its cysteine mutants.

## 1 Data description

The data presented in this article were obtained with the lipocalin allergen Mus m 1.0102 [[1], [2], [3]] and its cysteine mutants MM-C138A, MM-C157A and MM-C138,157A. The data derive from: 1) a Dynamic Light Scattering-based thermostability assay, 2) a Fluorescent-based ligand-binding assay and 3) an *in vitro* allergenicity test.

**Fig. 1:**
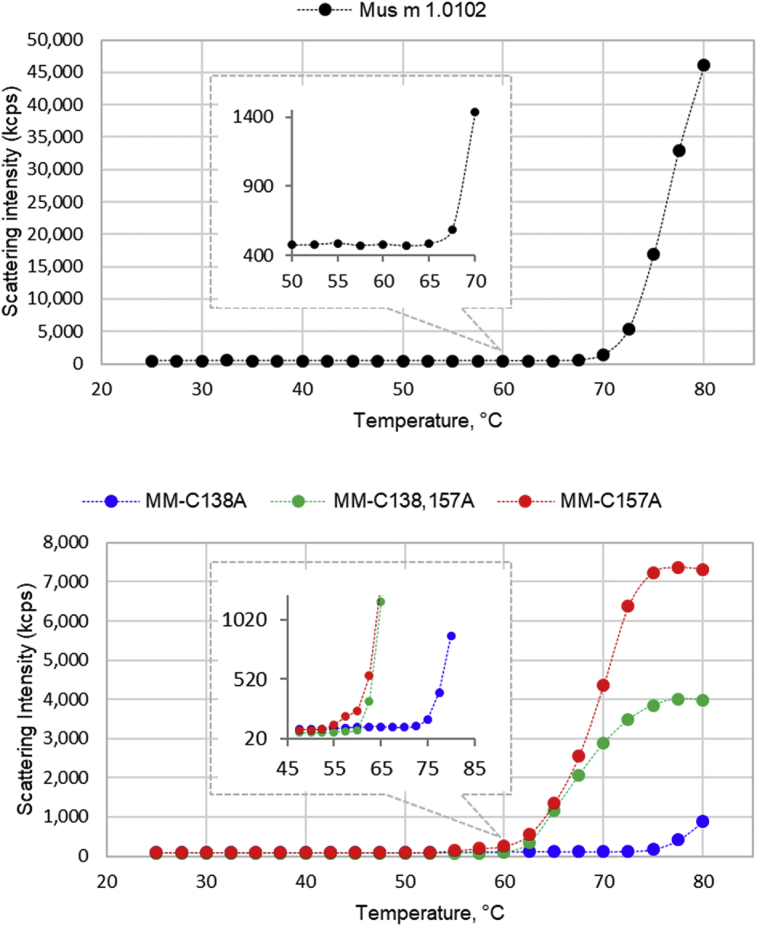
**Protein thermal unfolding monitored by dynamic light scattering.** Light scattering intensities of Mus m 1.0102 (A) and its mutants (B) are presented as a function of temperature. All the traces were generated by the scattering intensity parameter (kilocounts per sec, kcps). Malvern DLS dedicated software calculated the following temperatures, corresponding to the onset of the protein unfolding (Tm_onset_): 62.5 °C for Mus m 1.0102, 52.5 °C for MM-C157A, 55.0 °C for MM-C138,157A and 70.0 °C for MM-C138A.

Dynamic Light Scattering data [4] of protein solutions are presented as scattering intensities (kilocounts per sec) during a temperature ramp from 25 to 80 °C (Fig. 1), and report the protein transition from folded to unfolded state and aggregation. The temperature associated to an apparent signal increment (Tm_onset_) corresponds to the onset of the protein unfolding, being the temperature at which the protein hydrodynamic radius starts to increase. The Tm_onset_ values, presented in Fig. 1 caption, were calculated by a DLS dedicated software. Higher temperatures are indicative of aggregates formations.

**Fig. 2:**
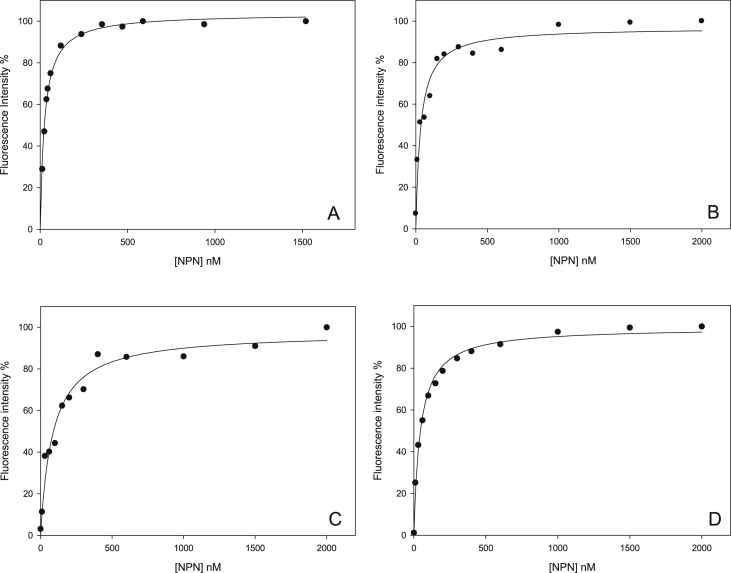
**N-Phenyl-1-naphthylamine binding to Mus m 1.0102 and its mutants.** Fluorescence direct titrations of recombinant Mus m 1.0102 (A), MM-C138A (B), MM-C157A (C) and MM-C138,157A (D) mutants with N-Phenyl-1-naphthylamine (NPN) fluorescent probe. Data are normalized with maximum fluorescence value set to 100%. Fluorescence data sets were analysed by nonlinear regression to calculate the following equilibrium dissociation constants ± SE: 25.0 ± 1.5 nM for Mus m 1.0102, 33.8 ± 6.4 nM for MM-C138A, 85.5 ± 13.7 nM for MM-C157A and 45.2 ± 4.0 nM for MM-C138,157A.

The ligand binding test was performed by means of direct titration experiments, by increasing additions of N-Phenyl-1-naphthylamine (NPN) to protein solutions; the intensity of fluorescence emission of NPN probe, bound to Mus m 1.0102 and its mutants, was measured (Fig. 2). Fluorescence intensity data sets [4] were analysed by nonlinear regression to calculate the equilibrium dissociation constants, Kd, presented in Fig. 2 caption.

**Fig. 3:**
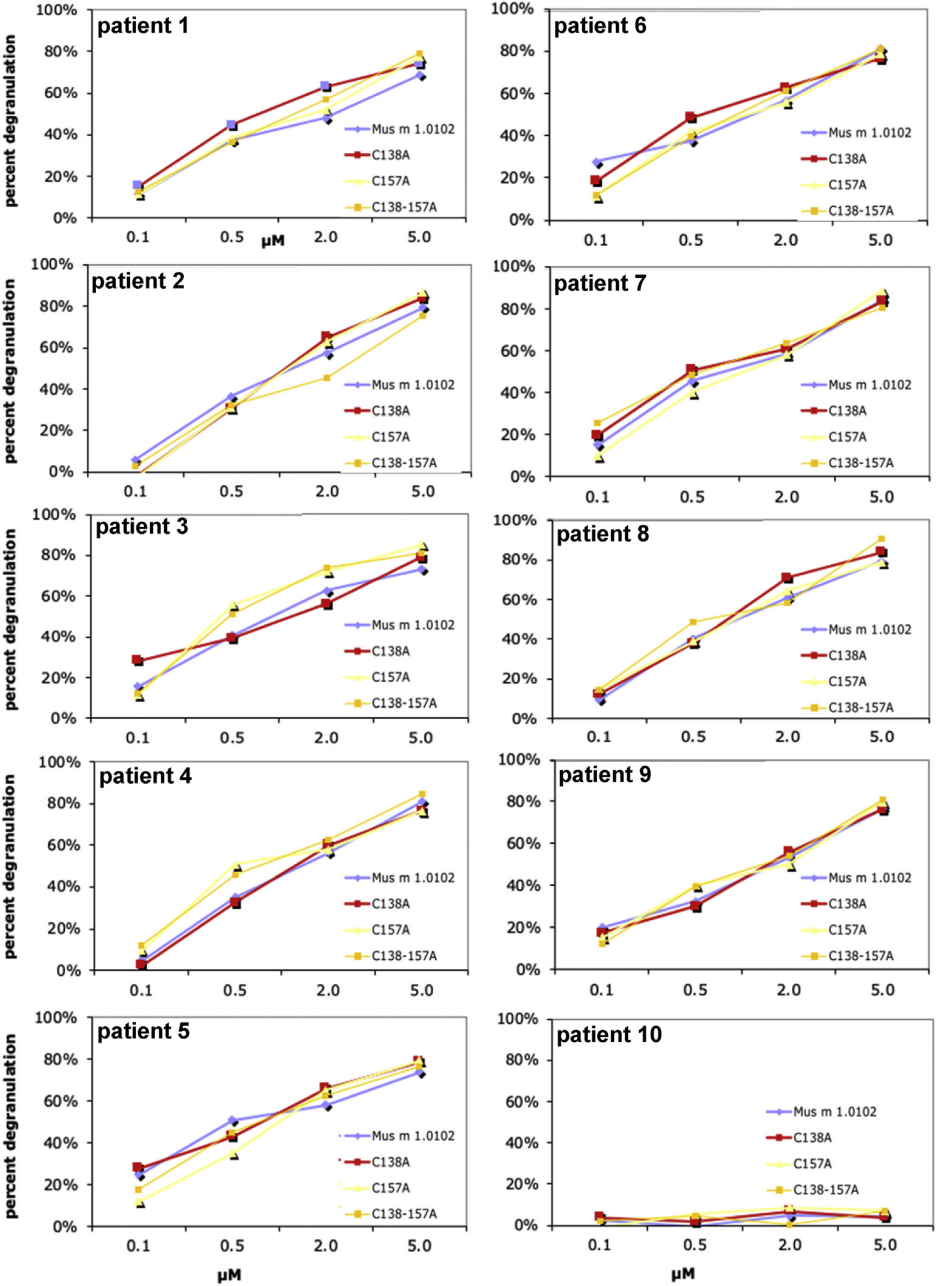
**IgE mediated degranulation.** IgE mediated degranulation of human FcεRI-expressing RBL-SX38 cells that were incubated with sera from ten allergic individuals (to load IgE to their IgE receptors) and challenged with either native Mus m 1.0102 or MM-C138A or MM-C157A or MM-C138-157A mutant. Degranulation was measured as percent of maximum β-hexosaminidase release (*y-axis*) upon incubation with different antigen concentrations (*x-axis*). Serum of patient 10, allergic to cat, dog and horse dander proteins, but not to mouse urinary proteins (Mus m 1 major allergen), confirms Mus m 1.0102-specificity of the data.

Table 1 reports the diagnosis and serum IgE concentrations of ten allergic patients, selected to assess the proteins’ allergenic potential. A degranulation assay was used as an *in vitro* allergenicity test. The assay used Rat basophilic leukemia cells, sensitized with the serum of the selected allergic patients, and measured the release of β-hexosaminidase in presence of increasing concentrations of Mus m 1.0102 and its cysteine mutants (Fig. 3) [4].

**Table 1:** Demographic and clinical features of the selected allergic patients.

ID	Age^a^	Sex^a^	Diagnosis^b^	years from diagnosis	Total IgE^c^	Mus m 1^d^	Fel d 1^e^	Equ c 1^e^	Can f 1^e^
1	20	m	RCA	7	88	1.35	0.99	0.00	1.10
2	19	m	RC	2	247	0.89	1.08	8.54	0.00
3	18	f	RC	11	159	5.90	4.03	0.00	0.54
4	30	f	RCA	6	204	0.82	12.11	0.00	4.21
5	21	f	RC	N.A.^f^	90	65.39	5.03	0.00	9.01
6	29	f	RC	5	77	5.13	0.59	0.00	0.00
7	23	f	RCA	3	123	7.11	1.56	0.00	1.24
8	28	m	RC	8	98	1.63	4.22	0.00	0.00
9	30	m	RCA	4	159	5.81	14.44	8.07	0.54
10	26	m	RC	8	357	0.00	22.45	12.00	2.12

a Numbers indicate age in years, F or M indicate sex.

b The clinically prevalent diagnosis is indicated (RC: rhinoconjunctivitis, A: asthma).

c Total IgE titer is expressed in International Units/mL (IU/mL).

d Mus m 1, Fel d 1, Equ c 1 and Can f 1-specific IgE are reported in ISAC Standardised Units (ISU, by ImmunoCAP ISAC Immunoassay, Phadia, Uppsala).

e Fel d 1, Equ c 1 and Can f 1 are, respectively, cat, horse and dog allergens.

f N.A.: not available in clinical records.

## 2 Experimental design, materials, and methods

### 2.1 Thermal stability assay

For this set of experiments, recombinant proteins were dissolved in Tris-HCl 10 mM pH 7.2 at concentrations ranging from 45 to 240 μM and filtered (Anotop siringe filter 0.02 μm, Whatman) before use. Measurements were carried out using a Malvern Zetasizer μV instrument, the *Temperature trend* measurement type and a 2 μL quartz cuvette (ZMV1002, Malvern).

*Thermal unfolding.* To detect the onset of thermal unfolding, experiments were carried out using an automated 2.5 °C incremental temperature ramp in the interval 25–80 °C, with a 60 s equilibration time at each measurement step. As measurement progresses, the instrument software interpolates the most recent six points of the intensity trace in the aggregation point graph (Fig. 1) and calculates the slope; the temperature at which the slope exceeds the value of 1 corresponds to the melting onset temperature (Tm_onset_).

### 2.2 Ligand binding assay

Direct titration experiments were performed with the fluorescent probe N-Phenyl-1-naphthylamine (NPN, Sigma Aldrich), an effective probe for Mus m 1 proteins since it becomes strongly fluorescent when bound inside their hydrophobic pocket [5]. Measurements were performed on a JASCO FP-6200 spectrofluorometer, equipped with a Peltier-controlled cuvette holder. NPN binding was monitored by exciting at 337 nm and recording the emission spectra over the range 350–550 nm. Protein solutions, 20 nM in TrisHCl 10 mM pH 7.2, were titrated by increasing additions of NPN (up to the final concentration of 1500 or 2000 nM); fluorescence intensity was measured at 25 °C, 20 min after the addition of NPN to allow for equilibrium to be reached. The per cent of maximum fluorescence intensity (recorded at *λ*_max_ = 401 nm) was plotted versus increasing NPN concentration and a nonlinear regression analysis (SigmaPlot v. 11, Systat Software) was applied to calculate the dissociation constants of Mus m 1.0102 and its mutants.

### 2.3 Criteria for allergic patient selection

Ten subjects, in the 18–30 age range, were selected for the *in vitro* allergenicity test.

All subjects fulfilled the following inclusion criteria: 1) they were diagnosed with allergic rhinoconjunctivitis with or without asthma by an experienced board-certified allergist, 2) their diagnosis was based on a clinical history of typical respiratory symptoms, appearing minutes or hours after exposure to the allergen source, according to the European Academy of Allergy and Clinical Immunology (EAACI) criteria, 3) minimum time from diagnosis was 2 years (only in one case this information was not available), 4) they had access to a standard pathogen-free animal facility for professional reasons.

Determination of allergen-specific IgE was performed by means of ImmunoCAP ISAC Immunoassay (Thermo Fisher/Phadia, Uppsala).

Patients 1–9 are allergic to Mus m 1, having Mus m 1-specific IgE titers in the range 0.82–65.39 ISU (ISAC Standardised Units); in addition, they have specific IgE molecules to at least one of the following allergens: Fel d 1, Equ c 1 and Can f 1.

Patient 10 was included as a control, being not sensitized against Mus m 1 allergen, but having Fel d 1, Equ c 1 and Can f 1-specific IgE molecules.

Informed consent for the use of sera was obtained from the allergic patients.

### 2.4 *In vitro* allergenicity test

*Degranulation assay.* Rat basophilic leukemia cells (RBL SX-38, a kind gift by M.H. Jouvin and J. P. Kinet, Department of Pathology, Beth Israel Deaconess Medical Center and Harvard Medical School, Boston, MA, USA), stably expressing human FcεRI α, β and γ chains, were cultured as previously described [6]. Following sensitization with IgE from a subset of human allergic donors, they were stimulated with allergen concentrations varying from 0.1 to 5.0 μM and a dose-response test of β-hexosaminidase release was performed [7].

*Experimental procedure and data analysis.* To measure the amount of β-hexosaminidase released from the cells after the incubation with the allergen, *p*-Nitrophenyl-*N*-acetyl-β-D-glucosaminide substrate (p-NAG, Sigma-Aldrich) in 0.1 M citrate buffer (pH 6.2) was added to RBL-SX38 cell supernatants and incubated at 37 °C for 120 min. The reaction was terminated using 0.1 M carbonate buffer pH 10 and the absorbance measured at 405 nm. The supernatant of non-stimulated cells was used as a negative control. The maximal stimulation was determined by using 100 ng of 4-hydroxy-3-nitro-phenacetyl (NIP)-Bovine Serum Albumine (stoichiometric ratio NIP:BSA >10) (Biosearch Technologies, Vacaville, CA) as an IgE cross-linker [8], which reacted with RBL cells pre-incubated with a commercially available human recombinant IgE anti-NIP monoclonal antibody (Serotec, Milan, Italy) [9]. Negative control values consistently fell in the range of 15–25% of the total β-hexosaminidase content, assessed from the 0.05% Triton X-100 (Sigma-Aldrich) lysate of the cell monolayer. The extent of allergen reactivity (allergenicity) was expressed as percent of β-hexosaminidase release according to the following equation:

100 x [A_405nm_Sample−A_405nm_ Negative Control]/ [A_405nm_ Maximal Stimulation− A_405nm_ Negative Control]

The authors ensure that the work described has been carried out in accordance with The Code of Ethics of the World Medical Association (Declaration of Helsinki) for experiments involving humans.
